# Multisensory Spatial Analysis and NDT Active Magnetic Method for Quick Area Testing of Reinforced Concrete Structures

**DOI:** 10.3390/ma16237296

**Published:** 2023-11-23

**Authors:** Paweł Karol Frankowski, Tomasz Chady

**Affiliations:** Faculty of Electrical Engineering, West Pomeranian University of Technology, Szczecin, ul. Sikorskigo 37, 70-313 Szczecin, Poland

**Keywords:** multisensory, spatial analysis MSA, multisensory transducer, nondestructive testing NDT, nondestructive evaluation NDE, reinforcement bars detection, rebars, concrete inspection, reinforced concrete

## Abstract

This paper aims to present multisensory spatial analysis (MSA). The method was designed for the quick, simultaneous identification of concrete cover thickness *h*, rebar diameter, and alloys of reinforcement in large areas of reinforced concrete (RC) structures, which is a complex and unsolved issue. The main idea is to divide one complex problem into three simple-to-solve and based on separate premises tasks. In the transducers designed with the MSA, sensors are arranged spatially. This arrangement identifies each RC parameter separately based on the different waveforms/attributes. The method consists of three steps. All steps are described in the paper and supported by simulations and statistical analysis of the measurement. The tests were carried out using an Anisotropic Magneto-resistance (AMR) sensor. The AMR sensors can measure strong DC magnetic fields and can be combined in spatial transducers because of their small size. The selection of the sensor was extensively justified in the introduction section. The spatial transducer and the identification’s simplicity can allow for high accuracy in the real-time area testing of all three parameters. The risk of misclassification of discrete parameters was strongly reduced, and the *h* parameter can be identified with millimeter accuracy.

## 1. Introduction

The evaluation of reinforced concrete (RC) structures by nondestructive testing (NDT) methods is of great practical importance. Reinforcement corrosion is a serious (multi-billion-dollar) issue and is only one of many possible problems [1,2].

The evaluation of RC constructions can be conducted in many different ways. The methods range from destructive to semi-destructive to completely nondestructive testing. Semi-destructive testing is a group of methods (e.g., pull-off, pull-out, or core tests) that do not endanger the structure. After the tests, the damage is repaired. In the case of destructive testing, the tested object, after the inspection, is no longer fit for service even if the object passes the examinations (e.g., 3-point bend test, tensile testing, or macro sectioning). The NDT methods are often preferred over semi-destructive and destructive because no damage is dealt to the object. These methods are usually also cheaper and faster than other techniques. Many different parameters of RC structures can be tested, including pressure exerted on formwork by concrete [3], porosity of the concrete [4,5,6], durability of the concrete [7], strength of the concrete [8], microcracking (caused by overloading, deficient design, and fatigue) [9,10], defects (such as voids, honeycombs and tendon ducts) [11,12], carbonation level of the concrete [13], concrete cover thickness [14,15,16], diameter of the reinforced bar (rebar) [14,15,16], alloy of the rebar [14,15,16], etc. This work focuses on the evaluation of reinforcement parameters using NDT. This problem was discussed many times, e.g., in [13,14,15,16,17,18,19,20,21]. Therefore, a detailed description is skipped in this work.

### 1.1. Abbreviations and Symbols Nomenclature

A large number of abbreviations and symbols are used in this article. The list of abbreviations and the abbreviation expansions are provided in Table 1.

The list of symbols is presented in Table 2.

### 1.2. Motivation

NDT methods (unlike the destructive and semi-destructive) can be easily used in many points of the tested object without causing damage to the structure or significantly increasing costs. Therefore, they better reflect the actual state of the facility. Unfortunately, even NDT methods in the vast majority can only be used for spot testing. A very small number of techniques can be used for quick testing of large areas in their entirety [13,14,15,16]. The need to develop area testing methods results from the specificity of reinforced concrete structures. Defects may appear in them locally under the influence of various factors. Therefore, the possibility of a quick examination of the entire facility (in order to locate the endangered area) is desirable. So far, no technique could make it possible on a large scale. The magnetic method is one of the most promising NDT techniques that can be used for this purpose. Practical, cheap, and fast area testing would be helpful in many different situations, and the scale of their application would be enormous. RC has been a dominant construction material for over a century. Many structures around the world built at the beginning of the twentieth century are still in service [17], and their service time is ending (RC structures are usually designed for 50–100 years [18]). Such objects required periodic inspections. The necessity of testing RC objects is imposed by law. Periodic inspections of the objects are regulated by a building code of a specific country and should usually be conducted once per five years. The area NDT tests may also find applications in the case of new structures, where acceptance tests are usually required. Such tests are conducted to determine if the requirements of a specification or contract are met [1,2,17,18].

So far, there is no effective method for quickly evaluating large areas of RC structures. However, the simultaneous identification of basic parameters such as diameter (*D*) and class of the rebars (the class defines the hardness and elasticity of the reinforcing steel) and the concrete cover’s thickness are also fraught with another problem. All three basic parameters affect the measurements’ results similarly, making simultaneous identification challenging and complex.

The paper aims to present Multisensory Spatial Analysis (MSA). The method’s main idea is to divide one complex identification problem into three simple-to-solve and based on separate premises tasks. It significantly simplifies the analysis and makes individual results independent of each other. The MSA can bring the magnetic technique to the method, allowing for a quick, cheap, and straightforward assessment of large surfaces.

### 1.3. Area Testing of RC Structures with NDT Methods

Many methods can be used to test RC structures. A review of such methods is presented in [13,14,15,19,20,21]. However, only electromagnetic and mechanical-wave systems can assess reinforcement grids directly (and effectively). Mechanical-wave techniques can be utilized to test many different properties of RC [13,22,23,24,25]. These techniques are well suited for testing concrete. However, in terms of reinforcement evaluation, they are inferior to electromagnetic/magnetic methods. The effectiveness of electromagnetic and magnetic methods results from the magnetic properties of reinforced concrete components. The DC or low-frequency AC electromagnetic field affects only steel bars (concrete is practically not interfering). It is a significant advantage over mechanical methods, where the waves are damped by concrete and reflected and dispersed by concrete additives (e.g., fine aggregate) or affected by voids. The methods that can be used for area testing include infrared radiation thermography (IR) with induction or microwave excitation, microwave methods like ground penetrating radar (GPR), radiography (X-ray), electromagnetic methods represented usually by the eddy current (EC) technique, and magnetic and capacitive methods. A comparison of these methods (in their most frequently considered variants) is presented in Table 3.

Infrared radiation thermography is an up-and-coming technique. The area testing comes easy. The basic procedure is to point the thermal imaging camera at the area of interest to read the thermal signature on the structure’s surface in real time. Unfortunately, this type of research requires prior heating (of the whole object or just the reinforcement grid) and cooling down phases. Currently, induction or microwave heating is often proposed for heating. Infrared thermography (IR) is mainly used to test concrete. The method can also be implemented for area testing, rebar detection, and even (under many conditions) to detect corrosion. The method’s effectiveness is strongly related to the concrete cover thickness. The IR can be used when the cover thickness is about 50 mm or lower. The long-time measurements and relatively low range are why this method is not commonly used in practice [13,26,27,28,29,30,31,32].

The GPR is the next up-and-coming method. This method is easy to use and can quickly scan large areas. Because the transmitter and receiver are in one housing, the technique requires only access to one side of the wall. The method is widely used in many area testing issues. In the case of RC structures, rebars can be detected from several centimeters up to ten or more meters (while other magnetic or electromagnetic methods usually have a maximum detection range below 200 mm). The method is well fitted to mapping multilayer reinforced meshes. GPR is also sometimes utilized to estimate the diameter of rebars, detect breaks and defects, or even detect corrosion (debonding). Unfortunately, the method has several disadvantages. The GPR is not effective if the concrete cover is thin. Factors like voids or variable internal moisture conditions may affect the results. Moreover, the GPR device is expensive, resolution is limited, and the results are difficult to interpret [13,33,34,35,36,37,38].

The range and resolution of radiography are very high. The method is designed for area testing and theoretically can be used (very effectively) to evaluate RC structures. The X-ray techniques enable (similarly to GPR or IR) indirect corrosion identification by detection of debonding [39]. However, there are several reasons why this method is rarely used to test RC structures. Usually, access to both sides of the tested element is required; the source and detector must be placed on both sides of the tested object. (This problem can be solved to some degree by back-scattering [40].) The inspections are time-consuming since there is a need to move the detector during the measurement. Moreover, tests of this kind may cause risks to human health, and the devices are expensive [13,41].

The EC tests can be used to localize rebars in the structure and precisely estimate basic structure parameters such as the thickness of the concrete cover, the rebar’s diameter, and the class of the rebar’s alloy. The effective range of the method is from 0 to even 100 mm. In some cases, the method can also detect corrosion or other flaws (reduction in diameter or irregularities in obtained signal) [42]. The method can be calibrated by adjusting the frequency. Low spatial resolution and extensive range are obtained using low frequencies of 10^2^–10^4^ Hz. On the other hand, excitation frequency at 10^6^–10^9^ Hz results in a high resolution and small range. It is assumed that the method can detect inhomogeneities of a size comparable to the wavelength of excitation [27,42,43,44,45].

The capacitive method is frequently used in rebar detection and concrete cover thickness identification. Generally, the method enables the identification of similar properties as the EC method. The bandwidth range is from 10^2^ Hz to 10^9^ Hz [46]. Sensors of this type usually can detect smaller inhomogeneities, but on the other hand, their effective range is also smaller than that of the EC or magnetic methods. Designing and assembling a sensor for area testing is easier in the case of this method than in the case of EC. Such a transducer would be also cheaper. Unfortunately, the method is sensitive to environmental pollution, and its range may be too small in some cases [47,48,49].

Magnetic methods can be divided into active, where an excitation is used, and passive (no magnetization device). The Magnetic Flux Leakage method (MFL) is the main representative of active methods. The MFL method may be used to localize rebars in the RC structure or detect rebar breaks and cracks. Other active magnetic methods (such as magneto-acoustic emission, Barkhausen emission, stress-induced magnetic anisotropy, and the magnetic powder method) are not frequently applied to evaluate RC structures. The active methods are more sensitive than the passive. However, this approach has some disadvantages, such as additional equipment and power consumption requirements. Passive methods are more economical and straightforward. Methods of this kind can be used to evaluate RC structures but are less effective than active ones [16]. One of the methods of this kind is the Magnetic Memory Method (MMM). The technique can detect abnormal conditions arising from changes in crystalline structures of rebar material resulting from stress concentration, corrosion, or cracks. The active magnetic method can be used similarly to the EC and capacitive techniques. The effective range in typical applications is comparable to the EC method, but the resolution is slightly lower. Magnetic sensors pose a high ability to perform area testing. Some elements, like Magneto-optical (MO) sensors, are designed and intended for area testing [14]. Others like the Hall effect element (Hall), Anisotropic Magneto-resistance (AMR), or Giant Magneto-resistance (GMR) sensors can be easily connected in the matrix. Moreover, the excitation system and data analysis are straightforward, making a magnetic method very cheap and applicable. The other advantage of magnetic testing is the possibility of analyzing particular spatial magnetic components. These properties create unique possibilities that no other technique gives. A more detailed summary of the EC, capacitive, and magnetic methods is presented in [6]. The magnetic method has a sufficiently large range and resolution, and its simplicity makes the construction of an area transducer straightforward and cheap. However, depending on the sensors used, the magnetic transducer for area testing can possess different properties [13,14,15,16]. Due to the rapid development of sensors, the method seems to have excellent development potential [50,51,52,53].

### 1.4. Selection of the Magnetic Sensor

The Magnetic Sensors Market size is estimated at USD 3.25 billion in 2023 and is expected to reach USD 4.76 billion by 2028, growing at 7.91% yearly. Magnetic sensors are widely used in NDT and new navigation devices, medical areas, presence detection in building automation-related applications, and the automotive sector. Sensors of this kind can use various physical phenomena such as Electromagnetic Induction, the Hall Effect, Tunnel Magneto-resistance (TMR), Giant Magneto-resistance, Anisotropic Magneto-resistance, and Giant Magnetoimpedance (GMI). Hall and magneto-resistance (MR) sensors comprise 98% of the magnetic sensor market. Over half of the market (in pieces) belongs to hall sensors, another 20% to AMR, about 5% to GMR, and 3% to TMR. The share of MR sensors in the market is increasing year by year [50,53]. In order to select sensors for the area testing transducer, it is essential to consider such parameters as the measuring range, bandwidth, and size of the sensor. A comparison of these parameters for the fundamental technologies of the sensors is shown in Table 4.

Solid-state Hall effect, GMR, TMR, AMR, and GMI sensors are manufactured using planar microfabrication processes and can offer high sensitivity and a compact size. Moreover, they are complementary to CMOS fabrication processes, making it feasible to integrate sensors with sensing and computing circuitry on a single chip [50] and enabling placing three sensors in one housing and measuring magnetic field induction spatial components. The electric and magnetic properties of AMR and GMR elements can be varied in an extensive range. The next advantage is low power consumption. In order to compare MR sensors, the following parameters should be considered: a magneto-resistance ratio, sensitivity, narrow hysteresis, low anisotropy field, large exchange bias field, minor changes in parameters with temperature, reliability, and repeatability. The GMR elements have advantages in these fields over the AMR sensor (and a high advantage over the Hall effect sensor). GMR can also be operated above the AMR sensor field range (Table 2).

On the other hand, AMR technology offers higher bandwidth (even 5 MHz for commercial solutions compared to 100 kHz for GMR) and can measure negative values (polarization recognition). Sensors of this kind also have a reset system that restores them to their original state after exceeding the measurement range. AMR technology also enables making sensors with a narrow detection range and a small hysteresis. Therefore, this technology is very well suited for high-precision applications (in contrast to GMR, where the high range comes with relatively high hysteresis). Moreover, other AMR sensor parameters are lower but still can be comparable to GMR. Currently, the measuring range of AMR sensors reaches up to a single Tesla, which makes them suitable for most commercial NDT applications [54].

Compared to MR elements, the Hall effect sensors have the least sensitivity, resolution, and other (mentioned earlier) parameters. However, elements of this kind can operate in strong magnetic fields, which is suitable for most industrial systems. The sensors can also offer a high dynamic range at a low cost. The low cost, adequate performance, and high availability make the Hall effect element the most prevalent element in the magnetic sensors market [50,51,52,55]. The bandwidth of commercial, silicon-based Hall effect sensors is usually limited from 0 to 10 kHz. The fastest elements of this kind available on the market can reach 1 MHz (untypical semiconductors are used) [56]. A comparison of the GMR, AMR, and Hall effect sensors is presented in Table 5.

Tunnel Magneto-Resistance sensors belong to the family of MR elements, and similarly to Hall, AMR and GMR are usually triaxial (three elements in one housing, each for another spatial component of magnetic induction). TMR is a relatively new technology (compared to GMR or AMR), characterized by low susceptibility to temperature changes (high stability), strong output, high magnetic sensitivity, high bandwidth, and high resistance. The high magnetic sensitivity and high resistance give them low power consumption and high accuracy. TMR elements are also relatively often combined into matrices or vectors to form area-testing transducers. Sensors of this kind can find applications in devices with limited power or technologies that demand high-accuracy control [50,51,52,56,57,58]. Sensors of this type are not designed to test strong magnetic fields. Therefore, they cannot be used to evaluate reinforcing bars.

Giant Magneto-Impedance sensors are the least common of the MR components. They are characterized by relatively large size, very high resolution (usually many times greater than other MR-type sensors), an extensive measurement range, a large bandwidth, and high dynamics. Despite numerous advantages, they do not have significant market shares, primarily because of the high price [50,51,52,55,59].

Magneto-optical sensors are designed and intended for area testing. The application of such elements to evaluating RC structures is presented in [14]. The MO-measuring system is relatively expensive but universal and easy to use. Elements of this kind are designed to detect relatively strong DC magnetic fields [14,55].

The fluxgate magnetometer (fluxgate sensors) is a magnetic sensor designed for a vector magnetic field. Sensors of this kind are large. Traditionally, it has been used as a compass or metal detector. The normal range is suitable for measuring the earth’s field and can resolve well below one 10,000th of that. Fluxgate sensors are much more sensitive than Hall sensors and have better temperature stability, low noise, and linearity. The main disadvantages are size and their small range of operation [50,55,60].

The highest sensitivity is obtained for superconducting quantum interference devices (SQUID). Sensors of this kind can reach sensitivity at fT (10^−15^ T) [43]. The elements of this kind require cooling down to very low temperatures, which causes high costs and complexity. They are also relatively large (only some of their components can be miniaturized) [50]. The bandwidth of these sensors can range from 0 to hundreds of MHz [61].

Induction sensors based on the coil are usually bulky and robust and find application in industrial sensing systems. Search coils have an extensive bandwidth and theoretical measurement range. These elements can be used only for AC magnetic fields. Sensors of this kind are not easily miniaturized due to the difficulty in fabricating 3D coils and the poor performance of planar coils compared to their wire wound counterparts. Moreover, the coil’s sensitivity is strictly connected with the number of turns (size of the element) [50].

To sum up, MO sensors can be used to test reinforced concrete structures. The significant advantage is that each film particle is like a separate sensor, so they are sold as ready-made area testing sensors. However, such systems are expensive and quite complicated. A more detailed description and analysis is presented in [14]. Sensors such as AMR, GMR, and Hall are relatively small. Therefore, it can easily be combined into arrays. In each of these groups, models that would be well suited for evaluating reinforcing bars can be found. Each of these groups of sensors has advantages and disadvantages compared to the two others, as described earlier. A measuring coil could also be considered. However, the element would have to be movable in such a system. Due to its large size, creating an array of sensors would be difficult. Such a system would also be much more complex than in the cases of AMR, GMR, or Hall sensors.

## 2. Measuring System, Samples and Methods

This section provides technical details about the measurements, the measurement system, and the methods used in the research.

### 2.1. Measuring System and Samples

RC structures are described by three primary parameters: concrete cover thickness *h*, rebar diameter *D*, and rebar class. The class parameter describes the rebar alloy’s mechanical properties (flexibility and hardness) [62]. The concrete cover thickness *h* usually ranges from 20 to 50 mm. In the experiments, this range was extended to 20–70 mm, with the step of 10 mm giving six instances: *h*_20_, *h*_30_, *h*_40_, *h*_50_, *h*_60_, and *h*_70_. In the samples, two rebar diameters (*D*_10_ = 10 mm and *D*_12_ = 12 mm) and three classes (AI—highest flexibility and lowest hardness of the alloy; AIII—low flexibility and high hardness; and AIIIN—lowest flexibility and highest hardness) are used. The utilized materials are commonly used and available at most building supply stores. The popularity of the materials utilized in the experiments makes the tests relevant to civil engineering reality. The list of used in the experiments rebars is presented in Table 6. Because concrete does not affect the results of magnetic measurements, its description is omitted.

**Figure 1:**
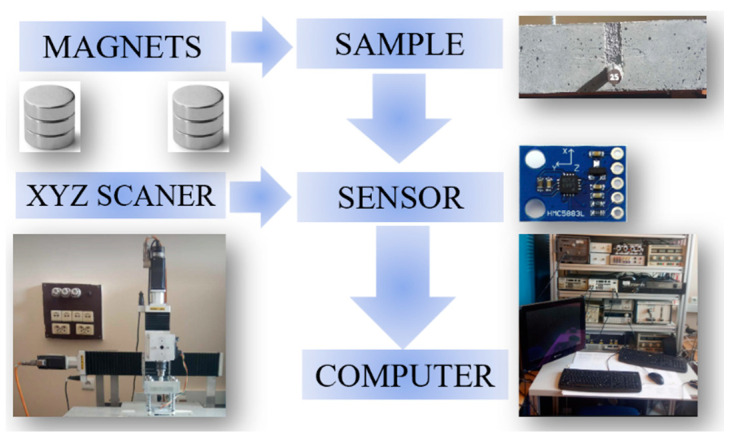
Block scheme of the measuring system [16].

One of the most significant advantages of the magnetic NDT method is simplicity. The measuring system is straightforward and consists of five parts: excitation subsystem, positioning subsystem, tested sample, magnetic field transducer, and data acquisition subsystem. The block diagram of the system is shown in Figure 1.

The excitation subsystem consists of two neodymium magnets, M1 and M2, placed on the sample’s surface directly above the rebar (Figure 1). Magnets M1 and M2 interact with the reinforcing bar in the sample, magnetizing it. The positioning subsystem (XYZ scanner) moves the sensor over the sample. The sensor moved (in a specific, programmed way) by the scanner measured the spatial components of the magnetic field induction (the sensor scans the sample). Then, the values measured by the sensor are sent to the measuring computer (the data acquisition subsystem).

**Figure 2:**
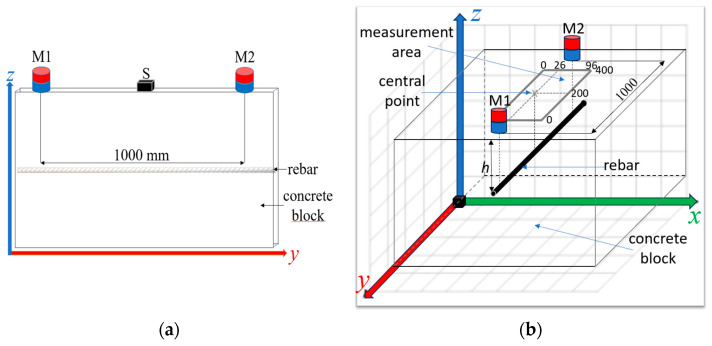
Schematic view of the sample and elements of the measuring system: M1 and M2—magnets; S—sensor (HMC5883L); SPM configuration. (**a**) 2D side view; (**b**) 3D view with depicted measurement area.

The advantages and disadvantages of the three magnetization methods versions were presented in the earlier work [16]. The same pole magnetization (SPM) configuration, where the magnets are orientated to the sample with the same poles, was chosen for the tests. Change from the typical opposite pole magnetization (OPM) to SPM significantly increases the signal amplitude, repeatability, and signal-to-noise ratio (SNR). The SPM magnetization is also more resistive for waveform shape deformations. (A more detailed comparison is presented in [16].) The magnets are static and not moved during the measurements. The distance between M1 and M2 equals 1000 mm (the distance was determined experimentally). In order to increase readability, the accurate scale in Figure 2 has not been kept.

The following criteria were established to select the appropriate sensor: measuring range of about 1 mT to 1 T, the ability to measure the DC magnetic field, and the possibility of building a transducer for area testing are coming from small size to connect sensors into a matrix or sensor designed for area tests. Other parameters were not of great importance (in this issue). Based on Table 4, these requirements can be met by some Hall effect, AMR, GMR, and MO sensors. The MO sensor and the AMR (the HMC5883L (Honeywell, Morris Plains, NJ, USA) [54]) were selected for the tests. (The results received for the MO sensor are discussed in [14]). The advantages of AMR over Hall and GMR sensors are described in the previous section. Elements of these kinds have many advantages over MO sensors. The most important is the possibility to measure magnetic field induction spatial components (*B*_x_, *B*_y_, and *B*_z_), system simplicity, low cost, and high sensitivity and field resolution.

Rebars with a diameter from 10 to 12 mm (*D*_10_, *D*_12_) are usually connected to a reinforced grid with 150 or 200 mm (150 × 150 or 200 × 200 mm) eyes. The measuring area was significantly limited to minimize the impact of adjacent rebars on the results. Due to the symmetry of the waveforms, the measurements on one side of the reinforcing bar were shortened. It allowed us to shorten the measurement time without significant loss of information. Measurements in the *y*-axis (*y*-scan) were made with steps of 10 mm (from 0 to 400 mm), and in the *x*-axis (*x*-scan), were made with steps of 1 mm in the range of 0 to 96 mm. The central point (in the middle of the rebar) is *x* = 26 mm, *y* = 200 mm (Figure 2).

### 2.2. Methods

#### 2.2.1. Boxplot Graphs

**Figure 3:**
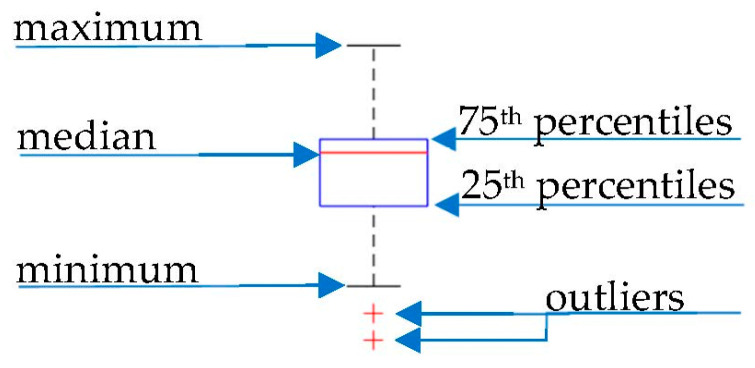
Description of the boxplot graph [16].

The boxplot graph in this paper presents the selected variable’s statistical distribution. The description of the boxplot is shown in Figure 3.

Each box’s bottom and top edges indicate the 25th and 75th percentiles, and the central mark indicates the median. The whiskers extend to the most extreme data points, not including outliers. The outliers are plotted individually using the ‘+’ marker symbol.

#### 2.2.2. Amplitude and Offset Calculations

**Figure 4:**
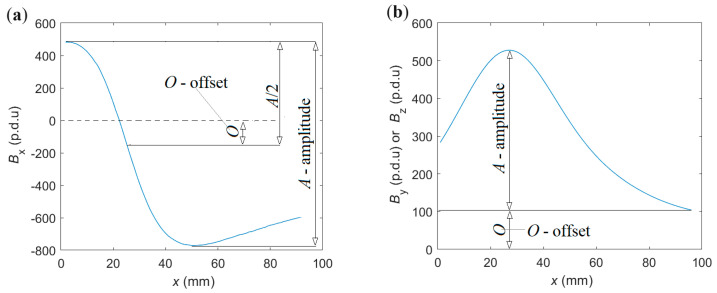
Definition of offset and amplitude: (**a**) *B*_x_, (**b**) *B*_y,_ and *B*_z_.

The definition of the parameters is presented in Figure 4.

This paper determines the amplitude as the difference between the largest and smallest measured waveform values. In the case of *B*_x_, the offset is given as maximum value minus minimum value divided by two (if the arrangement is fully symmetrical, it should equal the measurement value received directly above the reinforcing bar) or as a minimum value in the case of *B*_y_ and *B*_z_.

#### 2.2.3. Extraction of Attributes from Measured Waveforms

In [15], it was proposed to divide the attributes describing the waveform or signal into three categories:

1. Offset attributes (O)—the difference between the signal’s minimum (or average) value and the zero level. In the case of simple waveforms (such as those analyzed in this paper), it is a single attribute specified with a specific value. In more complex cases, these could be attributes describing, for example, a trend line showing how the offset changes over time.
2. Amplitude attributes (A)—it is essential to keep these attributes independent. In simple cases, an attribute in this category is the maximum value minus the minimum value (in this way, the offset is separate from the amplitude). In more complex cases, this category also includes local maxima, minima, points of inflection, etc.
3. Shape attributes (S) are many ways to describe the shape. The basic ones are described in [15]. The key is to maintain the independence of the attributes. The shape attributes should always refer to a normalized waveform and be independent of amplitude and offset.

The set of attributes based on which the analysis will be carried out should be selected based on three fundamental rules:

1. Attributes are as independent as possible.
2. The attributes accurately reflected the shape of the waveform/signal.
3. The number of attributes is as small as possible (avoiding the curse of dimensionality). Typically, one parameter should be identified based on one to five attributes.

Typically, rule #2 conflicts with rule #3. The fundamental problem must be faced when parameters are identified based on the waveform/signal. There are many methods to eliminate and merge attributes (reduce the number of attributes without significantly reducing considered information). The essential tools are wrappers and filters [63]. Selecting attributes is time-consuming, highly arbitrary, and requires experience to carry it out correctly. PCA and rough set theory are other, slightly more efficient, and less arbitrary methods. However, as the number of attributes is reduced, the waveform becomes less and less accurately reflected (in many cases, this problem can be solved by ACO decomposition [15]). This issue and possible ways to avoid this problem are discussed in more detail in [15]. In this work, the waveform is considered in terms of amplitude (A), offset (O), and waveform shape (S). At the same time, all three attribute extraction rules are maintained (mutual independence, small number, and good representation of the waveform shape).

## 3. Results

The first part of this section presents the expected benefits that may result from using sensor arrays. The expectations come from earlier conducted research [14,15,16]. Then, the results of numerical simulations are presented. The simulations are used to introduce the SPM method, verify measurement results, and present fundamental relations between the sample parameters and magnetic field distribution. The third subsection presents the relations between the parameters of RC structures and the obtained measurement results. Finally, the MSA method is presented, and specific solutions are proposed for identifying the three primary parameters of the RC structure.

### 3.1. Expected Advantages Resulted from the Use of Sensor Arrays

The use of multi-sensor transducers in area testing has many advantages, such as

1. Enabling the identification of three parameters based on different premises from several independent waveforms (and attributes extracted from them). This procedure significantly simplifies and increases the efficiency of identification. Instead of identifying many parameters based on a single waveform (one set of attributes), separating and isolating the specific tasks is possible. Then, each can be solved based on separate premises (separate sets of attributes). For example, one parameter is identified based on measurement along the *x*-axis, another based on measurement along the *z*-axis, etc;
2. Significant reduction in the measurement time (area testing instead of a time-consuming series of point measurements);
3. Adjusting the transducer setting to the unusual positioning of the rebar;
4. Simultaneous measurement in multiple places next to each other—reduction in the impact of noise by averaging the results;
5. Simultaneous identification of a single parameter based on various independent premises—increasing reliability and error detection.

Practically all the listed advantages of using one-, two- and three-dimensional arrays of sensors can be replaced with a scan in the appropriate number of dimensions. However, the scanning approach can cause a significant extension of the measurement time, the increased complexity of the measurement system, and the increase in costs.

### 3.2. Simulations

**Figure 5:**
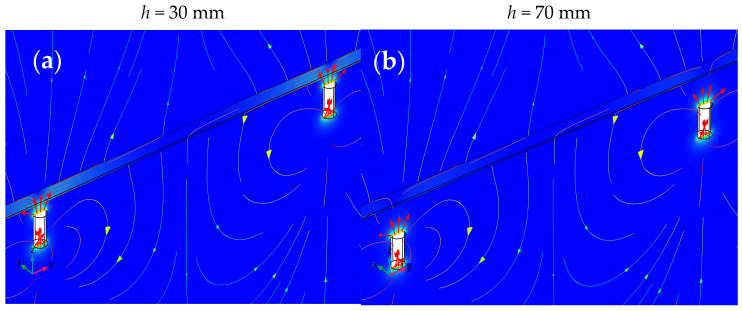
The simulation of the spatial distribution of normalized magnetic flux density lines received for two concrete cover thicknesses (*h*) and three magnetic permeability (*µ*). (**a**) *µ* = 100, *h* = 30 mm; (**b**) *µ* = 100, *h* = 70 mm.

Simulations are performed using the finite element method (FEM). For this purpose, COMSOL v6.0 software is used. The program works as the solver for the calculations and is utilized to present the results graphically. Normalized lines of the magnetic flux density are presented in Figure 5. The distribution of field lines is characteristic of a specific method of magnetization. In the case of normalized magnetic induction values, it can be observed that the distribution is very similar for different concrete cover thicknesses. The biggest differences can be observed right next to the magnets.

**Figure 6:**
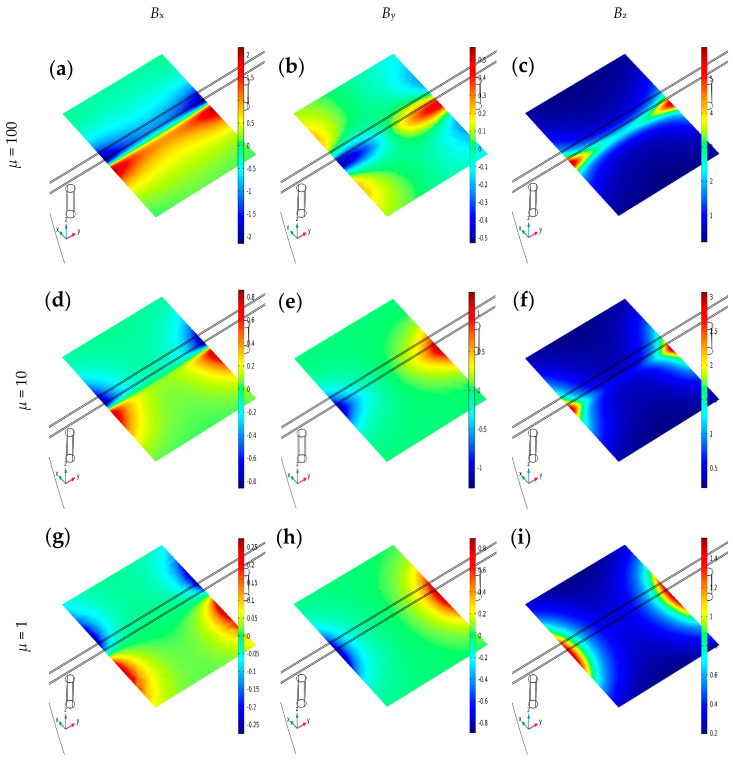
The magnetic flux density distribution simulations in the XY plane were obtained for different magnetic permeabilities. The rebar and two magnets are also presented in the visualization; *µ*, SPM and *h* = 30 mm: (**a**) *B*_x_, *µ* = 100, (**b**) *B*_y_, *µ* = 100, (**c**) *B*_z_, *µ* = 100, (**d**) *B*_x_, *µ* = 10, (**e**) *B*_y_, *µ* = 10, (**f**) *B*_z_, *µ* = 10, (**g**) *B*_x_, *µ* = 1, (**h**) *B*_y_, *µ* = 1, (**i**) *B*_z_, *µ* = 1.

The SPM method, apart from a stronger signal and lower noise level compared to typical OPM, is characterized by high repeatability and low variability of results [16]. The normalized distribution of magnetic flux density lines is invariant and characteristic of the magnetization method, as presented in [16]. The non-normalized values differ and mainly depend on the magnets’ strength, the tested object’s magnetic permeability (*µ*), and the distance between the magnet and the tested object (rebar). The impact of the magnetic permeability of the tested material on the spatial components of magnetic flux density is presented in Figure 6.

Simulations confirmed that the tested material’s magnetic permeability (*µ*) can strongly impact the results (all magnetic field induction spatial components). In the case of steel rebars, the greater the *µ*, the higher the magnetic flux density, and the stronger the signal received from the magnetic sensor (it can be assumed that *µ* of steel is 100). The value of the *µ* has the most impact on the *B*_x_ and the lowest on the *B*_y_. Increasing the *µ* value ten times, the *B*_x_ value increases by less than three times. In such a case, change in *B*_z_ increases about twice, while change in *B*_y_ is minimal. Further experiments have shown that, in most cases, the hardness of steel is correlated with its magnetic permeability.

**Figure 7:**
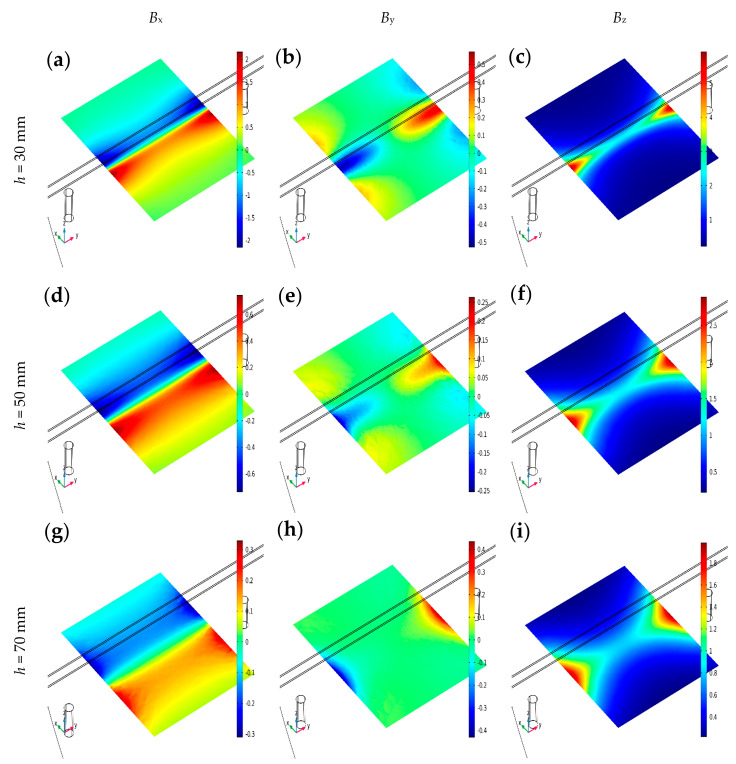
The magnetic flux density distribution simulations in the XY plane were obtained for different concrete cover thickness *h*, SPM, and *µ* = 100. The rebar and two magnets are also presented in the visualization; (**a**) *B*_x_, *h* = 30 mm, (**b**) *B*_y_, *h* = 30 mm, (**c**) *B*_z_, *h* = 30 mm, (**d**) *B*_x_, *h* = 50 mm, (**e**) *B*_y_, *h* = 50 mm, (**f**) *B*_z_, *h* = 70 mm, (**g**) *B*_x_, *h* = 70 mm, (**h**) *B*_y_, *h* = 70 mm, (**i**) *B*_z_, *h* = 70 mm.

The other important factor is the concrete cover thickness (*h*). The impact of the *h* on the measurement results is presented in Figure 7.

The simulations presented in Figure 7 prove how vital the thickness of the concrete cover is to the amplitude of the measurements. As *h* increases, the values of *B*_x_, *B*_y_, and *B*_z_ decrease, all in the same proportions. These changes are not proportional to the change in *h* (the relationship is not linear). The strongest signal is obtained for *B*_z_, followed by (with a slight difference) *B*_x,_ and the weakest for *B*_y_.

**Figure 8:**
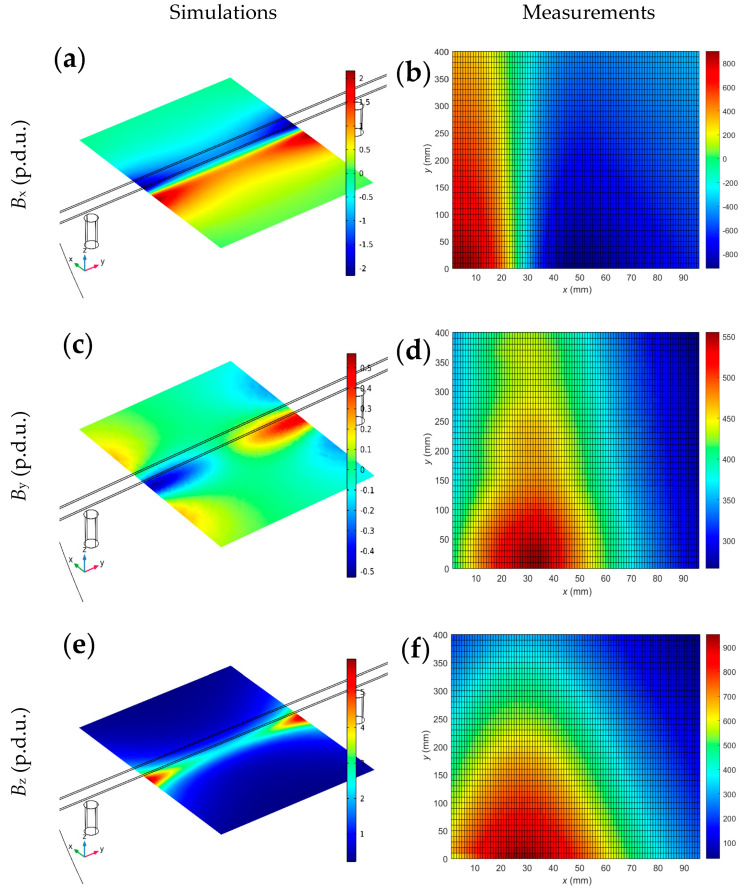
Comparison of the simulations and measurements; the magnetic flux density distribution in the XY plane for all spatial components, SPM magnetization, *h* = 30 mm, and magnetic permeability *µ* = 100. The rebar and two magnets are also presented in the visualization of the simulations; (**a**) simulation, *B*_x_, (**b**) measurement, *B*_x_, (**c**) simulation, *B*_y_, (**d**) measurement, *B*_y_, (**e**) simulation, *B*_z_, (**f**) measurement, *B*_z_.

Figure 8 shows a comparison between actual measurements and simulations. Measurement areas are much smaller than those considered in the simulations. Therefore, simulations are an excellent complement to actual tests.

The measured results are similar and consistent with the simulations (Figure 8). These similarities confirm the simulation model’s correctness and the lack of apparent errors and mistakes in measurements. Minor differences between the measurement results and simulations result from the lack of precise knowledge about the parameters of the tested reinforcement and from the impact of noise.

### 3.3. Relations between the Parameters of RC Structures and the Obtained Measurement Results

This section shows interesting and essential relations between the parameters of the tested RC structure and the parameters of the measured waveforms. Understanding these interactions can significantly simplify and increase the efficiency of identification. Most observations are the same for magnetic and electromagnetic (eddy current) methods. The section consists of three parts. Each subsection has a different RC structure parameter (*h*, *D*, class).

#### 3.3.1. Identification of the Concrete Cover Thickness

**Figure 9:**
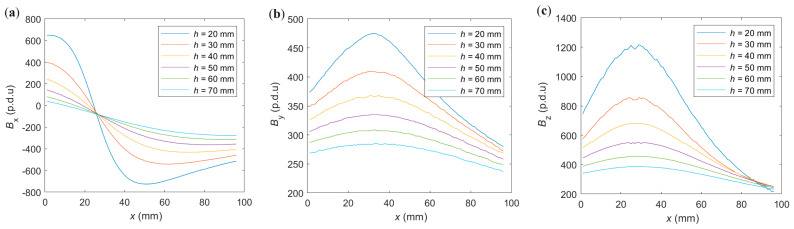
The measurements of spatial components of magnetic induction vs. *x* position, for six different sample concrete cover thickness, OPM magnetization, and *P*_2_ rebar: (**a**) *B*_x_, (**b**) *B*_y_, (**c**) *B*_z_.

The thickness of the concrete cover *h* is a continuous parameter. The identification of this parameter based on measurements is more straightforward than identifying the rebar diameter or class and can be made with millimeter accuracy [15,16,44]. The examples of measurements along the *x*-axis (*x*-scan) obtained for six different *h* (RAW data) are presented in Figure 9.

The obtained measurement results presented in Figure 9 confirm the conclusions from the simulations (Figure 7). As *h* increases, the values of all three spatial components of magnetic induction decrease, all in the same proportions. The strong signals are obtained for *B*_x_ and *B*_z_ and much weaker for *B*_y_.

**Figure 10:**
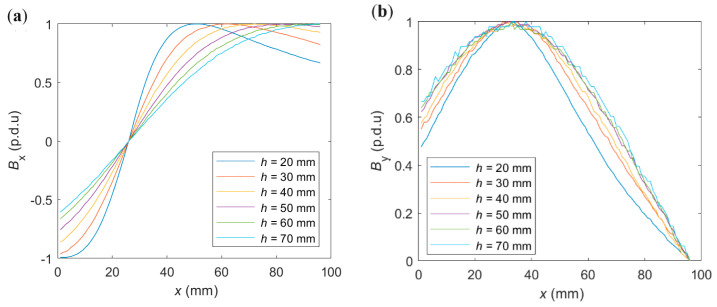

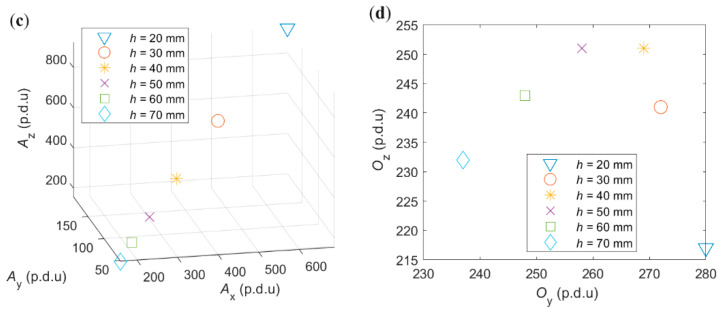
Measurements presented the impact of the concrete cover thickness *h* on the (**a**) shape of *B*_x_ component, (**b**) shape of *B*_y_ and *B*_z_ components, (**c**) amplitude (A), and (**d**) offset (O).

The changes in the thickness of the concrete cover affect all three types of attributes (A_x_, O_x_, S_x_) extracted from *x*-scans. Figure 10 shows the shapes of normalized waveforms. Shapes of the spatial components *B*_y_ and *B*_z_ obtained for different *h* are very similar. Therefore, only one of them is presented in Figure 10.

As shown in Figure 10a,b, the greater the thickness of the concrete cover *h*, the slower changes in the normalized waveform value in function of the sensor position (lower value of the derivative of the waveform concerning *x*).

In Figure 10a, it can be observed that the maximum value of the waveform changes position as the cover thickness changes. This property can be used in the identification process. Waveforms of this type are symmetrical. Therefore, the minimum value can be used similarly to the maximum.

Figure 10b shows that as the *h* increases, the differences between the waveforms decrease. At the same time, the noise level increases, which can make identification based on these forms much more difficult.

The offset in the *B*_x_ case is constant and equal to the value of the *B*_x_ measured directly above the rebar. For this reason, the offset value O_x_ for *B*_x_ (O_x_(*B*_x_)) is omitted in Figure 10d. The independence of the O_x_ attribute value from one of the parameters is a great advantage (over O_y_ and O_z_) that can be used in the identification process.

The O_z_ parameter does not change monotonically as a function of the *h*. For this reason, it cannot be used as the sole attribute based on which identification will be carried out.

**Figure 11:**
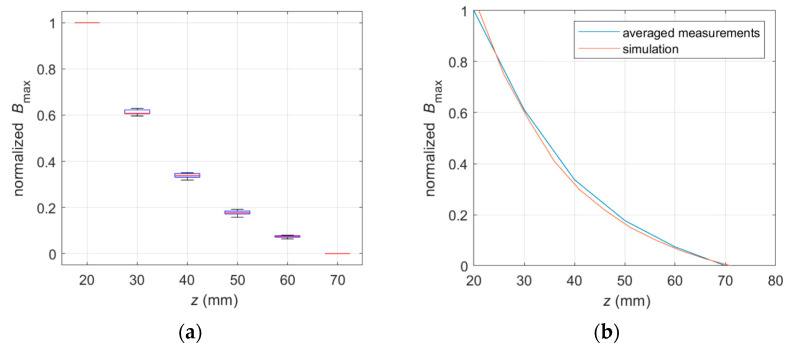
Measurement and simulations of the max. magnetic induction *B*_max_ as a function of the transducer position on the *z*-axis; curves obtained for four types of rebars (*P*_1_, *P*_2_, *P*_3_, and *P*_4_), three spatial components (*B*_x_, *B*_y_, and *B*_z_), and five sensor positions; central point and 10 mm and 20 mm from it in both directions. A total of sixty different measurements; (**a**) the boxplot presents the repeatability of measurements and (**b**) comparison of averaged measurements results to the simulations results.

The shape of the curve representing the maximal value of the magnetic induction *B*_max_ in the *h* function (Figure 11) is identical for spatial components (*B*_x_, *B*_z_, and usually *B*_y_ [16]; *B*_y_ is not recommended to be used in the identification process [16]). For this reason, the relation presented in Figure 10c is linear. Moreover, the shape of the curve presented in Figure 11 is independent of every parameter of the system or tested sample (the rebar diameter, the class of the steel, the method of magnetization, or the kind of spatial component in analysis), except the distance of the rebar from the sensor. This independence is crucial to the efficiency of the identification process.

The waveforms obtained for different samples differ very slightly (Figure 11a). The repeatability of measurements is also high. The obtained measurement results do not differ significantly from the simulation results (Figure 11b). All this makes the waveform presented in Figure 11 very well fitted for identifying the cover thickness.

With at least three sensor array layers, it has become possible to identify the concrete cover’s thickness independently from other parameters with millimeter precision (as presented in Figure 11). Solving this problem requires solving only equations with three unknowns (1).

(1) $$\left\{\begin{array}{c} B_{1}=A\cdot f\left(h\right)+O\\ B_{2}=A\cdot f\left(h+n\right)+O\\ B_{3}=A\cdot f\left(h+m\right)+O \end{array}\right.$$

where *B*_1_—result of the measurement (single sensor) in the first layer of the sensors; *B*_2_—result of the measurement (single sensor) in the second layer of the sensors (directly above first sensor); *B*_3_—result of the measurement (single sensor) in the third layer of the sensors (directly above second sensor); *A*—amplitude (unknown); *O*—offset (unknown); *h*—concrete cover thickness/level of the first layer of the transducers (unknown); *n*—distance between first and second layer; and *m*—distance between first and third layer of the sensors.

A similar relation to that presented in Figure 11 was also observed for identifying the parameters of reinforcing bars using the eddy current method [44].

The shape attributes S_z_ of the curve presented in Figure 11 can be effectively determined by the interpolation method discussed in [15].

#### 3.3.2. Identification of the Rebar Diameter

The diameter of the rebar is a discrete parameter. Identifying *D* is the most difficult of all three fundamental parameters of the RC structure [44]. Two different values of *D* are tested in the experiments: *D*_10_ = 10 mm and *D*_12_ = 12 mm. The rebar diameter affects all attributes extracted from the scans along the *x*-axis (S_x_, A_x_, O_x_) and *y*-axis (S_y_, A_y_, O_y_). It can also impact the amplitude and offset of the waveform obtained from the scan along the *z*-axis (A_z_, O_z_). However, the shape of this waveform (S_z_) does not change regardless of the diameter of the reinforcing rebar.

**Figure 12:**
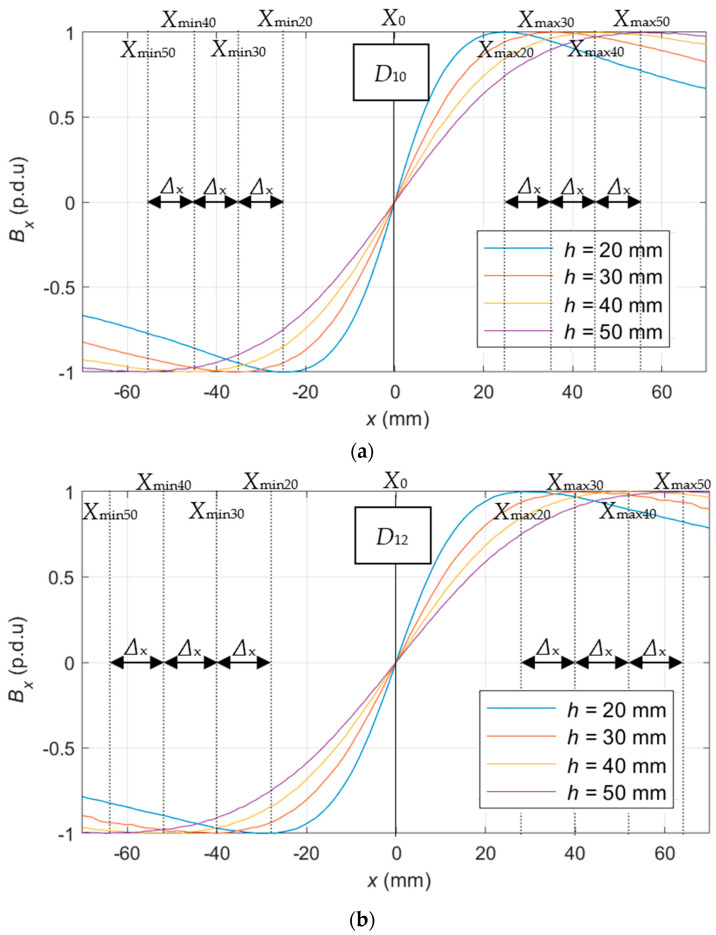
Extraction of shape attributes (S_x_) from the measurements of *B*_x_ waveform, using the characteristic points method [15]; identification of the rebar diameter; two types of attributes Δ_x_ and *X*_max_/*X*_min_; extraction carried out for (**a**) *D*_10_ and (**b**) *D*_12_.

In order to extract the shape attributes S_x_ from waveform *B*_x_ (S_x_(*B*_x_)), the characteristic points method (discussed in [15]) is recommended. Two types of such attributes are presented in Figure 12.

The *X*_max_ is a group of attributes showing what position on the *x*-axis corresponds to the maximum amplitude of the waveform for specific *h*, e.g., *X*_max20_ is the distance between position zero *x* = 0, and *x* position of maximal value of the waveform obtained from the measurements received for *h* = 20 mm. The Δ_x_ is the difference between subsequent *X*_max_, e.g., Δ_x_ = *X*_max30_ − *X*_max20_, or Δ_x_ = *X*_max50_ − *X*_max40_.

The position of the maximum value *X*_max_ changes with the increase in the concrete cover thickness (the sensor movement along the *z*-axis). This change is linear in function of *z*, and experiments show that it depends on the rebar diameter. Therefore, the Δ_x_ value (defined in Figure 12) is constant and is approximately equal to *D* per 10 mm (where *D* is the rebar diameter). Since the waveform is symmetrical, an analogous relation can be observed for *X*_min_. The Δ_x_ attribute fits the rebar diameter identification well because its value is independent of the sensor’s position on the *z*-axis and the rebar class. Unfortunately, near the maximum value of the waveform is a flattening, and noise can significantly affect the attribute value. In order to reduce the influence of noise, the measurement can be performed several times, in several places at the same time, or both (positive and negative) halves of the waveform can be used. The average value and standard deviations (δ) of the shape (S_x_) attributes are presented in Table 7.

**Figure 13:**
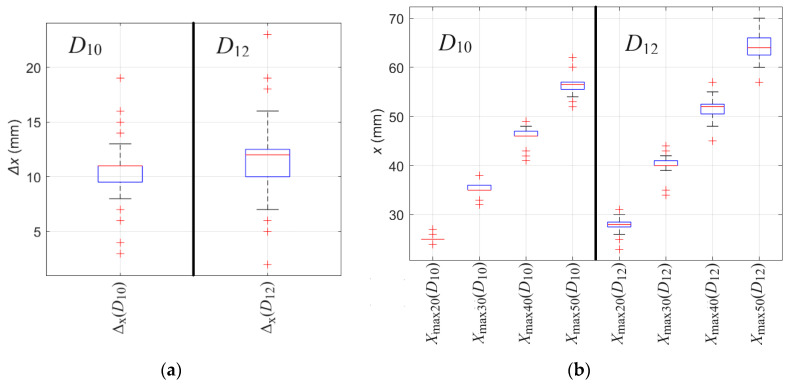
The boxplot presents the value repeatability of shape attributes S_x_, extracted from measurements of *B*_x_ spatial component of magnetic induction obtained from scanning along *x*-axis. The outliers are plotted individually using the ‘+’ marker symbol. (**a**) Δ_x_, (**b**) *X*_max_.

The *X*_max_ attributes, unlike Δ_x_, are not independent of the thickness of the concrete cover. However, as the distance between the sensor and rebar increases, the difference between attribute values obtained for different rebar diameters also increases. Therefore, the impact of the noise can be much less significant in this case. A more accurate representation of the statistics is shown in Figure 13. The statistical analysis of S_x_ attributes is performed based on twenty measurements for each diameter.

Several observations can be made based on Figure 13. Noise can strongly impact the value of both attributes Δ_x_ and *X*_max_. In the case of Δ_x_, a significant difference can be observed between Δ_x_ obtained for *D*_10_ and Δ_x_ obtained for *D*_12_. In the case of *X*_max_, the value changes linearly together with the concrete cover thickness. The greater the *h*, the easier it is to distinguish the rebar diameter (differences between Δ_x_ obtained for *D*_10_ and *D*_12_ are cumulative).

**Figure 14:**
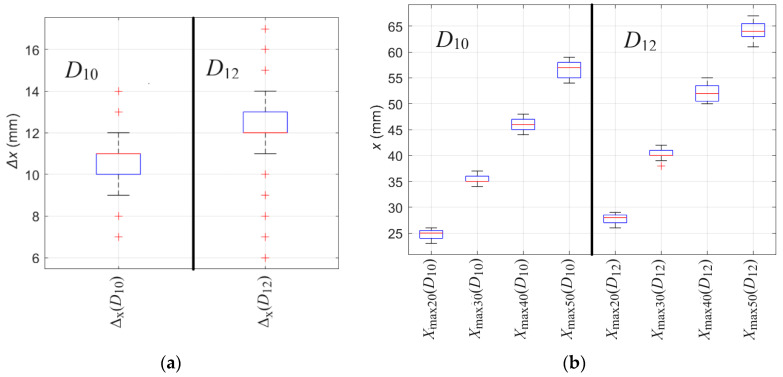
The boxplot presents the value repeatability of shape attributes S_x_, extracted from measurements of *B*_x_ spatial component of magnetic induction obtained from scanning along *x*-axis after the filtration. The outliers are plotted individually using the ‘+’ marker symbol. (**a**) Δ_x_, (**b**) *X*_max_.

Identifying the diameter of a rebar is a relatively difficult task. The essential element of identification is to reduce the influence of noise. As previously mentioned, the impact of the noise can be reduced statistically by using multiple measurements. Another way is signal filtering. Using a simple median filter with a window of five allowed us to improve repeatability and remove most outliers significantly. The median filter runs through the waveform element (entry) by element (over the entire signal), replacing each one with the median of five neighboring entries (window). The window is the first two preceding and two following entries. The filter allows the removal of outliers and smoothing of the waveform. Additionally, the method is straightforward and does not introduce the risk of strong distortion, smoothing of sharp edges, or excessive waveform interpolation (the filter only slightly modifies the waveform). The results obtained after the filtration are presented in Table 8 and Figure 14.

Based on Figure 14, it can be observed that using a simple median filter reduced the deviation and the number of outliers significantly. In the case of Δ_x_(*D*_10_), the range decreased from 16 (3–19) to 7 (7–14), and in the case of Δ_x_(*D*_12_), the range decreased from 23 (1–24) to 11 (6–17). Moreover, outliers were almost completely removed in all cases of *X*_max_.

**Figure 15:**
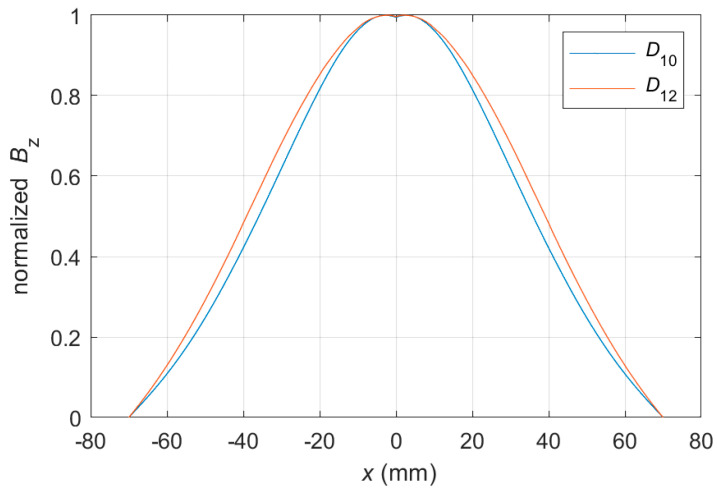
Waveforms obtained for measurement of *B*_z_, *x*-scan, *h* = 30 mm, and two different diameters of rebar: 10 and 12 mm.

The identification of rebar diameters can also be based on the *B*_z_ spatial component. A comparison of the waveforms obtained for rebars with different *D* is shown in Figure 15.

The *B*_z_ waveform (scanned along the *x*-axis) has the highest signal-to-noise ratio (SNR) of all spatial components [16]. However, in the case presented in Figure 15, the differences between the shapes of the waveforms obtained for rebars of different diameters are minimal. Therefore, such identification would be much less specific. Moreover, as presented in Figure 10b, the greater the concrete cover thickness *h*, the greater the noise and the more complex the identification. The worst possibility is identification based on *B*_y_. The difference between the waveforms would be minimal (similarly in the case of *B*_z_), but the SNR would be much lower (lowest SNR), making such identification very uncertain. Moreover, *B*_z_ may be susceptible to deformation [16].

In the case of *D* identification, similarly to *h* identification, the presented relations coincide with those observed for electromagnetic tests using the eddy current (EC) method. The waveforms obtained from EC differential transducers resemble those presented in Figure 12. Waveforms from absolute transducers are very similar to those presented in Figure 15. The relations between waveforms and RC structure parameters are also very similar [44].

#### 3.3.3. Class of the Rebar Identification

The class of the alloy from which the rebars are made is a discrete parameter. However, this parameter may sometimes be considered continuous since rebars from different manufacturers may have different electromagnetic properties (standardization only for mechanical properties) for unknown alloys. Then, the following relation can usually be observed: the harder the steel, the greater the magnetic permeability. The greater the magnetic permeability of the rebar, the stronger the signal in magnetic and electromagnetic investigations (this effect is shown in the simulations section and in [16]). More pronounced differences in class identification can be observed using OPM magnetization [16]. However, in this research, the classes are known (the parameter is discrete). Three different classes are tested in the experiments: AI—flexible and soft, AIII—inflexible and hard, and AIIIN—the hardest and most inflexible.

**Figure 16:**
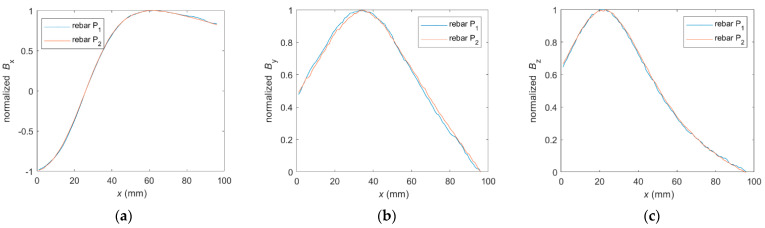
Normalized waveforms (coming from the measurements) obtained for different classes (P_1_-AI, P_2_-AIIIN) and different spatial components of magnetic induction: (**a**) *B*_x_, (**b**) *B*_y_, and (**c**) *B*_z_.

The most characteristic property of the steel alloy (as a parameter under identification) is that the class does not affect the shape of the standardized waveform. This phenomenon was observed in both magnetic and EC experiments [44] and is presented in Figure 16.

The use of reinforcing bars with the same physical properties but from different suppliers may result (during magnetic measurements) in obtaining different amplitude and offset values. However, the shape of the waveforms will not change (Figure 16).

### 3.4. Multisensory Spatial Analysis (MSA) in Use to Identification of RC Structures

Making transducers in the form of sensor arrays has many advantages. Not only does it significantly speed up the measurement or perform many measurements side by side simultaneously (which helps reduce the influence of noise) but this arrangement also allows for the proper arrangement of the transducer in XYZ space (if the rebar is positioned unusually). Positioning of the transducer is crucial. (All the analyses presented so far will not work if the transducer is positioned concerning the rebar at an unusual angle.) Even if it is possible to automatically recalculate the measurements and correct errors from incorrect transducer positioning, this would require a dense network of sensors and complicate the analysis. Moreover, in this case, area testing is even more needed. However, the most significant advantage of this type of transducer is that it enables the identification of various parameters to be independent of each other.

The MSA is intended to enable the spatial arrangement of sensors in the transducer so that during measurements, each of the RC parameters of the structure could be identified separately based on the different premises. For this purpose, measurements along three dimensions (XYZ) are first conducted (*x*-scans, *y*-scans, and *z*-scans). Next, it is necessary to find waveform parameters independent of some (in best cases, all besides one) sample parameters. Errors in the identification usually result from the fact that the value of a specific attribute does not depend only on the value of one specific parameter (simultaneous identification of three parameters), e.g., using a sample with a reinforcing bar of a larger diameter, a more flexible alloy and a smaller concrete cover thickness, a very similar waveform can be obtained as in the initial case. The purpose of MSA is to avoid this type of error by making the identification processes of individual parameters independent.

The analysis should be performed separately for each spatial component. The dependencies for the *B*_x_ are presented in Table 9.

The identification of each of the basic parameters of the RC structure can be performed based on the *B*_x_ component. It also provides opportunities that *B*_y_ and *B*_z_ do not offer. The thickness of the concrete cover is identified first. In order to obtain independence from other structure parameters, following Table 9 and Figure 12, the identification should be based on the shape attributes obtained from the *z*-scan (S_z_(*B*_x_)).

Once the cover thickness is known, the value of the *x*-scan shape attributes (S_x_(*B*_x_)) depends only on the rebar diameter (Figure 14). Only in the case of *B*_x,_ is it possible to determine Δ_x_ attributes independent of the cover thickness (although quite susceptible to noise). Additionally, the *X*_max_ and *X*_min_ attributes can be used. These attributes make verifying and correcting the *D* and *h* parameters possible.

The spatial component *B*_x_ has certain advantages over the other components when identifying the reinforcing bars. Theoretically, this identification can be based on any amplitude or offset, but in the case of *B*_x_, the offset obtained with *x*-scans (O_x_(*B*_x_)) is independent of the concrete cover thickness *h*, which is unnecessary but may be considered as an additional advantage. If the identification process is based on the O_x_ attribute, it deepens the independence of identification of individual parameters of RC structures.

Additionally, it should be noted that identification based on amplitude or offset is less precise than based on waveform. Magnetic sensors are manufactured in a specific sensitivity range. A slightly different amplitude may be obtained (the shape will remain unchanged) from different elements of this same kind. The amplitude and offset are also influenced by the precise positioning of the sensor between the magnets, the strength of the magnets, and the steel alloy from which the reinforcing bars are made (may depend on the supply and supplier). All of these factors are irrelevant to the shape attributes (S).

The *y*-scans are not used in the analysis. These scans have no unique advantages over *x*- and *z*-scans and are also the most susceptible to various factors, as discussed in [16].

The dependencies for the spatial component *B*_y_ are presented in Table 10.

The *B*_y_ spatial component is characterized by the weakest signal and the lowest SNR [16]. It makes possible the identification of the *h*. However, the results are uncertain due to the high impact of noise. Moreover, due to the shape of the waveform, the identification of *D* is much more complex than in the case of *B*_x_. This component also has no advantage in terms of identifying the class.

The dependencies for the *B*_z_ are presented in Table 11.

The strength of the signal and the highest SNR are the most significant advantages of *B*_z_ [16]. For these reasons, this spatial component of magnetic induction is best suited for identifying the thickness of the concrete cover. Other than that, it is not much different from *B*_y_.

## 4. Conclusions

The experiments show that many benefits may come from arranging sensors in XY and XZ planes (layered transducers). The layered arrangement of transducers allows the change of a complex and complicated problem (the simultaneous identification of three parameters) into three straightforward and easy-to-solve tasks.

If the transducer contains at least three layers of sensor arrays, it is possible to calculate easily the concrete cover’s thickness independently from other parameters and very accurately (millimeter precision).

The layered transducer also allows for the straightforward identification of the rebar diameter *D* (with high accuracy). The identification in the case of *D* is based on entirely different premises (waveforms and attributes) than in the case of *h* (measurement along the *x*-axis; *x*-scan). Therefore, an incorrect identification of *h* will not cause an incorrect identification of *D*.

The experiments prove that many factors can influence the amplitude and offset of the waveforms. Therefore, the *h* and *D* analyses are based on normalized waveform shapes. Very few factors influence the shape attributes and are much more stable.

In the case of identifying the class of reinforcing bars, the task is simplified because the remaining parameters have been identified earlier. This parameter does not affect the shapes of any waveforms and may be identified based on any amplitude or offset. The *B*_x_ offset has a significant advantage over other parameters because if independent of *h*.

Tests have shown that layered, spatial transducers (due to the simplicity of the calculations) can be used for area testing of large surfaces of RC structures. Such tests could be quick, and calculations can be performed in real-time.

The obtained results are auspicious, but further research is necessary. Situations where the reinforcing bar is placed at an unusual angle to the transducer may cause many additional problems. Confirming the method’s effectiveness also requires tests on a more extensive and more diverse number of samples.

Shortly, it is planned to conduct further analyses to search for other relations that could be used to identify the three primary parameters of the reinforcement. It is also considered to build a three-layer transducer for area testing and check its operation in practice.

Spatial transducers can be widely used in periodic control tests and inspections of newly constructed buildings. They can enable quick and straightforward checking of reinforcement parameters throughout the facility in a completely non-invasive way. Currently, no technology would make this possible.

## Figures and Tables

**Table 1:** Abbreviations and abbreviations expansions used in the paper.

**General:** RC—Reinforced Concrete, NDT—Nondestructive Testing.	**Methods:** MSA—Multisensory Spatial Analysis, IR—Infrared Radiation (thermography), GPR—Ground Penetrating Radar, X-ray—Radiography, EC—Eddy Current, MFL—Magnetic Flux Leakage, MMM—Magnetic Memory Method, SPM—Same Pole Magnetization, OPM—Opposite Pole Magnetization, ACO—Amplitude–Correlation–Offset (decomposition), SNR—Signal-to-Noise Ratio, PCA—Principal Component Analysis, FEM—Finite Element Method.
**Sensors:** MO—Magneto-optical, Hall—Hall effect element, MR—Magneto-resistance, AMR—Anisotropic Magneto-resistance, GMR—Giant Magneto-resistance, TMR—Tunnel Magneto-resistance, GMI—Giant Magnetoimpedance, SQUID—Superconducting Quantum Interference Devices.	
**Elements of the system:** S—AMR sensor (HMC5883L), M1—Magnet 1, M2—Magnet 2.	

**Table 2:** Symbols used in the paper.

**General:** *B*_x_, *B*_y_, *B*_z_—magnetic field induction spatial components, *B*_max_—maximal value of the magnetic induction (waveform), *µ*—magnetic permeability of the object. **Parameters of the RC structures:** *h*—concrete cover thickness (mm), * h*_20_:*h* = 20 mm, * *… * h*_70_:*h* = 70 mm. *D*—diameter of the rebar (mm), * D*_10_:*D* = 10 mm, * D*_12_:*D* = 12 mm, class—rebar alloy’s mechanical properties (flexibility and hardness), * *AI—highest flexibility and lowest hardness of the alloy, * *AIII—low flexibility and high hardness, * *AIIIN—lowest flexibility and highest hardness.	**Attributes:** O—Offset attributes * *O_x_—Offset attributes obtained for measurement along *x*-axis, * *O_x_(*B*_x_), O_x_(*B*_y_), O_x_(*B*_z_)—O_x_ obtained for specific spatial components of magnetic induction, * *O_y_, O_y_(*B*_x_), O_y_(*B*_y_), O_y_(*B*_z_)—similarly to O_x_, * *O_z_, O_z_(*B*_x_), O_z_(*B*_y_), O_z_(*B*_z_)—similarly to O_x_, A—Amplitude attributes * *A_x_, A_x_(*B*_x_), A_x_(*B*_y_), A_x_(*B*_z_), A_y_, A_y_(*B*_x_), A_y_(*B*_y_), A_y_(*B*_z_), A_z_, A_z_(*B*_x_), A_z_(*B*_y_), A_z_(*B*_z_)—similarly to O, S—Shape attributes * *S_x_, S_x_(*B*_x_), S_x_(*B*_y_), S_x_(*B*_z_), S_y_, S_y_(*B*_x_), S_y_(*B*_y_), S_y_(*B*_z_), S_z_, S_z_(*B*_x_), S_z_(*B*_y_), S_z_(*B*_z_)—similarly to O, *X*_max_—position of the *B*_max_ for S_x_(*B*_x_) * X*_max20_:*X*_max_ for *h* = 20 mm, * *… * X*_max70_:*X*_max_ for *h* = 70 mm. Δ_x_—difference between subsequent *X*_max_, e.g., Δ_x_ = *X*_max30_—*X*_max20._

**Table 3:** A comparison of the electromagnetic NDT methods for area testing.

Factor	IR	GPR	X-ray	Capacitive	EC	Magnetic
Resolution	•	•	•••••	••••	•••	••
Range	•	•••••	••••	•••	••••	••••
Low cost	•••	•	•	•••••	••••	•••••
Simplicity of use as area testing	•••••	•••••	•••••	•••	••	••••
Simplicity of measurements	•••	•••••	••••	••••	•••	••••
Fast area testing	••••	•••••	•••	•••	•••	•••
Simplicity of results interpretation	•••	•	•••••	•••	•••	•••
Simplicity of probe mounting	•••••	••••	•••	••••	•••	•••
Small number of limitations	•••••	••	•	•••	••••	••••
High safety of measurements	•••••	•••••	•	•••••	•••••	•••••
Work in dirty environments	••••	••••	•••••	••	••••	•••••
Work with thin materials	•••••	•	•••••	••••	•••	••••
Material Versatility	••••	•••••	•••••	•••	••	•

•••••—Best; ••••—Good; ••• —Medium; ••—bad; •—worst.

**Table 4:** Comparison of magnetic field sensors and their approximate measurement ranges, sizes, and bandwidths. The linear scale is changed to dB according to the formula *y* = 20·log_10_ *x.*

	Measuring Range (T)		Bandwidth (Hz|dB)		Size (m)
	from	to	from	to	
Coil	No limit		AC		10^−2^
Hall	10^−2^	10^2^	DC	10^5^|100	10^−6^
MO	10^−4^	10^2^	DC		10^−2^
GMR	10^−8^	10^2^	DC	10^5^|100	10^−6^
AMR	10^−10^	1	DC	10^7^|140	10^−6^
Fluxgate	10^−13^	10^−2^	DC	10^3^|60	10^−2^
TMR	10^−12^	10^−3^	DC	10^7^|140	10^−6^
GMI	10^−12^	10^−2^	DC	10^9^|180	10^−3^

**Table 5:** A comparison of AMR, GMR, and Hall effect sensors.

Factor	GMR	AMR	Hall
Resolution	•••	••	•
Low cost	••	•	•••
Physical size	•••	••	•••
Signal level	•••	••	•
Measurement range	•••	•••	•
Upper measurement limit	•••	••	•••
Lower measurement limit	••	•••	•
Power consumption	•••	•	•••
Temperature stability	•••	••	•
Narrow hysteresis	•••	••	•
Fit for precise applications	••	•••	•

•—worst; ••—medium; •••—best.

**Table 6:** Parameters of the rebars used in the experiments.

	*P*_1_	*P*_2_	*P*_3_	*P*_4_
*D*	10	10	12	12
Class	AI	AIIIN	AIIIN	AIII

**Table 7:** The average value and standard deviation of the S_x_ attributes extracted from measurements along *x*-axis.

	Δ_x_		*X*_max20_		*X*_max30_		*X*_max40_		*X*_max50_	
	*D*_10_	*D*_12_	*D*_10_	*D*_12_	*D*_10_	*D*_12_	*D*_10_	*D*_12_	*D*_10_	*D*_12_
mean	10.43	12.03	25.15	27.75	35.30	40.15	46.05	51.60	56.45	63.85
δ	2.87	3.65	0.81	1.74	1.45	2.39	2.04	2.60	2.35	3.03

**Table 8:** The average value and standard deviation of the S_x_ attributes extracted from measurements along *x*-axis after the filtration.

	Δ_x_		*X*_max20_		*X*_max30_		*X*_max40_		*X*_max50_	
	*D*_10_	*D*_12_	*D*_10_	*D*_12_	*D*_10_	*D*_12_	*D*_10_	*D*_12_	*D*_10_	*D*_12_
mean	10.55	12.13	24.90	27.90	35.30	40.10	46.05	52.10	56.55	64.30
Δ	1.58	1.99	0.85	0.85	0.86	1.07	1.15	1.65	1.54	1.87

**Table 9:** Relation between value of tested parameters and value of specific attributes obtained for *B*_x_ (analysis is based on measurements) [15,16].

	*B*_x_								
	*x* -Scan			*y* -Scan			*z* -Scan		
	A_x_	O_x_	S_x_	A_y_	O_y_	S_y_	A_z_	O_z_	S_z_
*h*	+	-	+	+	+	+	+	+	+
*D*	+	+	+	+	+	+	+	+	-
Class	+	+	-	+	+	+	+	+	-

‘+’—clear relation ; ‘-’—no relation.

**Table 10:** Relation between value of tested parameters and value of specific attributes obtained for *B*_y_ (analysis is based on measurements) [15,16].

	*By*								
	*x* -Scan			*y* -Scan			*z* -Scan		
	A_x_	O_x_	S_x_	A_y_	O_y_	S_y_	A_z_	O_z_	S_z_
*h*	+	+	+	+	+	+	+	+	+
*D*	+	+	+	+	+	+	+	+	-
Class	+	+	-	+	+	+	+	+	-

‘+’—clear relation ; ‘-’—no relation.

**Table 11:** Relation between the value of tested parameters and value of specific attributes obtained for *B*_z_ (analysis is based on measurements) [15,16].

	*Bz*								
	*x* -Scan			*y* -Scan			*z* -Scan		
	A_x_	O_x_	S_x_	A_y_	O_y_	S_y_	A_z_	O_z_	S_z_
*h*	+	+	+	+	+	+	+	+	+
*D*	+	+	+	+	+	+	+	+	-
Class	+	+	-	+	+	+	+	+	-

‘+’—clear relation ; ‘-’—no relation.

## Data Availability

Data available on request.
